# Time course of indirect reply comprehension in the young and older adults: an event-related potential study

**DOI:** 10.3389/fpsyg.2025.1578192

**Published:** 2025-07-10

**Authors:** Wangshu Feng, Xiaokun Zhang, Weijuan Wang, Lin Fan

**Affiliations:** ^1^Artificial Intelligence and Human Languages Lab, Beijing Foreign Studies University, Beijing, China; ^2^National Research Centre for Foreign Language Education, Beijing Foreign Studies University, Beijing, China; ^3^School of Foreign Languages, Qingdao University of Science and Technology, Qingdao, China

**Keywords:** pragmatic inference, cognitive aging, ERP, N400, working memory

## Abstract

**Introduction:**

In verbal communication, speakers often use implicit utterances that need listeners to interpret via pragmatic inference. As individuals age, their ability to make efficient and accurate pragmatic inferences may decline, leading to communication difficulties.

**Objectives:**

This study examined the cognitive aging phenomenon of pragmatic inference and its neural mechanisms using EEG recordings.

**Methods:**

Participants were presented with dialogues involving direct and indirect replies and were required to judge the speaker's intended meaning based on the context.

**Results:**

Younger participants outperformed older participants in task accuracy for indirect replies. Both groups exhibited an increased N400 for indirect replies in the late stages of reply presentation. However, younger participants exhibited greater N400 effects in the early and middle stages, something notably absent in older participants.

**Conclusion:**

These findings suggest that older adults have a reduced ability to make pragmatic inferences, likely due to difficulties in dynamically enriching semantic content in real-time speech.

## 1 Introduction

Currently, the global population is undergoing a remarkable aging trend, marked by a rapid rise in elderly population (Harper, 2014). As individuals age, structural and functional changes become widely distributed across numerous brain areas involved in various cognitive domains (Bagarinao et al., 2018; Campbell and Schacter, 2016; Gunning-Dixon et al., 2009). Language is a complex faculty that places substantial demands on humans' cognitive systems. Aging brings about declines in multiple language abilities, such as word production (Gordon and Kurczek, 2014; Yang and Zhang, 2019), phonological processing (Taler et al., 2010; Diaz et al., 2014), and semantic processing (Boudiaf et al., 2018; Zhu et al., 2019). In daily communication, listeners are tasked with interpreting not just the literal meaning of words but also extracting contextual hints to infer the speaker's meaning (Grice, 1975; Holtgraves, 1998; Hagoort and Levinson, 2014). This involves understanding the attitudes, emotions, and intentions behind the speaker's utterances, which is known as pragmatic inference. The decline in pragmatic inference ability can thus reduce an individual's communication skills and desire, thereby profoundly impacting their quality of life. Numerous neuroimaging studies have demonstrated that pragmatic inference requires the cooperation of linguistic and non-linguistic networks (Hagoort, 2013; Hagoort and Levinson, 2014). The neural correlates involved in this process include the dorsomedial prefrontal cortex (dmPFC), temporo-parietal junction (TPJ), insula, and inferior frontal gyrus, as well as middle/superior temporal gyrus, suggesting that successful pragmatic inference recruits the activation and integration of more fine-grained and broader semantic knowledge, in addition to inferring the speaker's mental state (Bašnáková et al., 2014; Feng et al., 2017, 2021; see Hauptman et al., 2023 for a meta-analysis).

Previous research has investigated the real-time processing of pragmatic inference during conversational contexts by using event-related potential (ERP) technology. Coulson and Lovett (2010) created short story scenarios ending in a 7-word target utterance (e.g., “my soup is too cold to eat”) that could be interpreted either literally or as an indirect request according to its context. From the perspective of the entire sentence, long-epoch ERPs revealed a sustained positivity for indirect requests, relative to the literal statements. When each word was compared with its own baseline, indirect requests triggered greater late positive components (LPC) than literal statements at the second (“soup”) and third words (“is”), while literal statements triggered a greater N400 effect than indirect requests at the fifth word (cold). These findings suggest that social context information can exert an initial impact on sentence processing, with the processing of indirect requests placing a higher cognitive load on memory retrieval. Another line of studies explored pragmatic inference by comparing indirect replies with direct replies. In these studies, the identical critical utterance (e.g., “I | really | have too many”) could be interpreted as either an indirect reply or a direct reply according to its preceding context. The ERP results indicated that indirect replies elicited an enhanced N400 at the final phrase (Guo et al., 2023; Zhang et al., 2023), suggesting more difficulty in semantic processing, and also showed enhanced sustained positivity during the final phrase (Zhang et al., 2021) or late negativity in the second and final phrase (Zhang et al., 2023), which may be linked to executive control or pragmatic reanalysis. As can be observed, ERP components related to pragmatic inference are inconsistent across various studies, which might stem from the usage of diverse experimental materials and the selection of baseline windows for analysis.

Age-related shifts in language comprehension have been investigated in healthy populations, and found to be well-preserved in behavioral performance (Shafto and Tyler, 2014). Meanwhile, measurements using ERPs in semantic processing have revealed differences between the younger and older adults with relative consistency, which demonstrates a decline in ERP. A collection of researches carried out by Federmeier and colleagues assessed how older and younger adults process words in sentential contexts online (Federmeier and Kutas, 2005; Federmeier et al., 2010; Payne and Federmeier, 2018; Wlotko et al., 2012). The findings revealed that older adults exhibited reduced predictive processing relative to their younger counterparts, as evidenced by smaller and later N400 responses to semantically incongruent or unexcepted words in older adults than in young adults, which suggested that older adults are less likely to utilize contextual content to guide semantic access of single words during sentence comprehension. Moreover, behavioral studies have highlighted the significant impact of age on pragmatic inferential processing during language comprehension, such as appreciations of novel metaphors (Mashal et al., 2011), proverbs interpretation (Uekermann et al., 2008), idiomatic ambiguity resolution (Grindrod and Raizen, 2019), sarcasm detection (Phillips et al., 2015; Rothermich et al., 2021), non-literal compliment interpretation (Pomareda et al., 2019), and humor comprehension (Baraldi and Domaneschi, 2024; Uekermann et al., 2006). Overall, older adults face challenges in accurately and efficiently interpreting non-literal language. A recent study has shed light on the factors contributing to failed pragmatic inference, and found that deficits in executive functions or theory of mind are predictive factors that can lead to age-related declines in communicative-pragmatic abilities (Bambini et al., 2021).

As examined in clinical populations, the difficulty in pragmatic inference can be attributable to various individual differences, such as deficient theory-of-mind (ToM) and executive dysfunction including low working memory (WM) capacity and poor inhibitory control (Bosco et al., 2017; Martin and McDonald, 2003; Ouerchefani et al., 2024). ToM processing has been naturally linked to pragmatic comprehension because understanding the speaker's meaning entails inferring her/his attitudes or intentions in a particular context (Carston, 2004; Sperber and Wilson, 1986). This link has been supported by evidence showing that the typical ToM network, encompassing dmPFC and TPJ, was activated during the processing of pragmatic inference (Hauptman et al., 2023; Yang et al., 2019). More importantly, inhibition of right TPJ activity via brain stimulation could interfere with indirect reply comprehension by reducing individuals' ToM ability (Feng et al., 2021).

Furthermore, studies involving individuals with neurological disorders or brain injuries also reveal associations between WM, inhibitory control, and various forms of non-literal language comprehension (Amanzio et al., 2008; Ferstl et al., 2005; Lacroix et al., 2024; Schettino et al., 2010). WM is considered as the cognitive processes and system that temporarily keeps information available. It is vital for language comprehension, enabling efficient short-term retention, manipulation, and integration of linguistic information. ERP studies have purposefully examined the influence of WM capacity on semantic integration at sentence and discourse levels (Ding et al., 2023; Van Petten et al., 1997; Yang et al., 2020). The results have consistently showed that the enhance of N400 effect to semantically anomalous or weak-predictable words was exclusively observed in the group with high (or medium) WM capacity; it was not detected in the group with low WM capacity. This pattern held true for both sentence and discourse contexts, and indicated that high WM capacity could facilitate the processing of semantic activation and integration utilizing contextual information. Additionally, Zhang et al. (2021) has investigated the impact of individuals' WM capacity on indirect reply comprehension. Participants with high and low WM capacity were exposed to dialogues featuring direct and indirect replies, and their brain responses were measured. The findings revealed that individuals with high WM capacity showed enhanced P200 (within the time window of 180–300 ms), P300 (300–800 ms), and LPC (800–1300 ms) responses to indirect replies compared to direct ones, suggesting earlier detection and processing of indirect meanings. In contrast, participants with low WM capacity only exhibited a delayed LPC effect, indicating later pragmatic analysis of indirect replies. This suggests that the WM capacity of individuals is crucial for the initial and immediate processing of pragmatic information.

Inhibition is also a key component of executive functions, which are essential for enhancing goal-directed behavior. Prior research has shown the integral role of inhibitory control abilities in language comprehension, including comprehending sentences, selecting the appropriate words, and managing multiple languages in bilingual individuals (Ye and Zhou, 2009). More importantly, Channon and Watts (2003) examined pragmatic inference using a social judgment task in adult participants (18–60 years old) that had suffered closed head injuries and control participants. They found individuals with closed head injuries exhibit deficits in interpreting indirect speech acts, as well as in performing the non-social executive tasks of measuring WM, inhibition and multitasking. Further regression analysis indicated that it was only inhibition, not WM or multitasking, that contributed significantly to explain the difficulty in pragmatic language comprehension. However, in healthy aging, working memory emerged as a robust predictor across multiple pragmatic tasks, while inhibition did not explain changes in those pragmatic tasks (Bambini et al., 2021).

In addition, educational attainment has been shown to be a robust predictor of baseline cognitive performance in reasoning and processing speed (Tucker-Drob et al., 2009). This observation aligns with Stern's (2002) conceptualization of preserved differentiation, wherein early-life cognitive advantages—shaped by enriched educational experiences—persist across the lifespan, maintaining relative performance disparities in later adulthood. Also, empirical evidence has shown that socioeconomic status broadly influences cognitive aging (e.g., Steptoe and Zaninotto, 2020; Migeot et al., 2022). Thus, in investigations of pragmatic aging—a process inherently tied to contextual language processing and social cognition—factors such as educational attainment and socioeconomic status must be considered, as they may confound or modulate observed neural and behavioral outcomes.

Although pragmatic inference has been extensively investigated through electrical recording and neuroimaging techniques, the neural substrates of age-induced changes in this cognitive process remains under-explored. Therefore, the current study aims to (1) explore whether there is an aging effect in pragmatic inference, (2) determine what the neural correlates might reflect about this aging phenomenon, and (3) identify what cognitive factors could help explain this aging phenomenon. To this end, we carried out an ERP experiment to investigate the real-time processing of indirect reply comprehension in both older and young adults. In this experiment, we used materials in written conversational dialogues, and manipulated the level of contextual relevance between a given reply and its context to form three experimental conditions: direct reply condition, moderately indirect reply condition, and highly indirect reply condition. Reply sentences are presented phrase by phrase, and the EEG data throughout the entire sentence presentation was recorded and analyzed. To account for the impact of individual differences as above-mentioned, we further collected demographic variables from the participants, including their educational attainment and subjective socioeconomic status. Additionally, we employed a series of well-established experimental tasks to assess various cognitive and social abilities of the participants. We used the Flanker task to assess participants' inhibitory control ability (Eriksen and Eriksen, 1974). By comparing reaction times between congruent (surrounding arrows aligned with the central arrow) and incongruent conditions (surrounding arrows pointed in the opposite direction), we evaluated participants' ability to inhibit irrelevant information. Greater reaction time delays in incongruent conditions compared to congruent conditions indicate a weaker ability to inhibit irrelevant information. Secondly, we used the digital span task to measure participants' working memory capacity (Wechsler, 1997). In this task, participants are asked to recall sequences of digits that increase in length. The score is determined by the maximum number of digits correctly recalled. Lastly, we used the comic completion task (Enrici et al., 2011) and the autism spectrum quotient (AQ) questionnaire (Baron-Cohen et al., 2001) as representative tools for evaluating participants' ToM ability. In the Comic Completion task, participants are required to choose the correct fourth panel of a comic strip from two options based on the content of the first three panels. The task includes intentional trials (where the correct option conveys a communicative intention through gestures) and non-intentional trials (where the correct option illustrates a physical causality between events). The greater the differences in accuracy and reaction times between intentional and non-intentional trials, the weaker the individual's ability to infer others' intentions from non-linguistic cues. The AQ questionnaire is a widely used self-report measure designed to assess traits associated with autism spectrum conditions. Higher total scores on the AQ indicate greater levels of autistic traits and lower social proficiency.

Based on foregoing research, we predicted that older adults, compared with young adults, would have difficulty in comprehending indirect replies. Neurally, indirect replies, compared with direct replies, would elicit enhanced N400 effects and larger late positive or negative components for certain words within the replies. More importantly, differences in age groups may be reflected in the reduction of the N400 effect, which is triggered by indirect reply comprehension, in the elderly group.

## 2 Materials and methods

### 2.1 Participants

Twenty-three university students at Beijing Foreign Studies University and twenty-two elderly people living in the community of Beijing Foreign Studies University were recruited for this study. Data from four participants were deleted due to extremely low accuracy in the reading comprehension task (1 elder), or low EEG data quality (2 young and 1 older adults). Ultimately, behavioral and EEG data were gathered and examined from a group of twenty elderly individuals (14 females, aged 57–78, with a mean age of 67.4 ± 7.6 years) and twenty-one younger participants (18 females, aged 18–23, with an average age of 19.0 ± 1.6 years). Among them, five participants were left-handed (3 participants in the older group and 2 in the young group). We evaluated the general cognitive state using the mini-mental state examination (MMSE, Folstein et al., 1975; Katzman et al., 1988), with all participants' scores being greater than 24. All participants were native Chinese speakers and had normal or corrected eyesight, with no history of hearing impairments, neurological issues or psychiatric conditions. Prior to the experiment, participants had signed an informed consent form and received a certain amount of compensation after the conclusion of the experiment. All experiments in this study was conducted with the approval of the Ethics Committee of Artificial Intelligence and Human Languages Lab, Beijing Foreign Studies University, China (#2020-0908-01).

We performed a series of age-related difference tests between the older and younger adults (see Table 1). The educational levels were similar for both groups (*W* = 206, p = 0.924). The subjective socioeconomic status measured by the single-item Subjective Socioeconomic Status Scale (Kilpatrick and Cantril, 1960) exhibited a marginally lower trend in the elderly compared to the younger adults (*W* = 139.5, *p* = 0.058). The WM span was significantly lower for the elderly, relative to the younger adults (*W* = 30.5, *p* < 0.001). For the flanker task, accuracy rates were high for both older and younger adults, with no significant differences observed between these two groups. ANOVA for mean RTs revealed significant main effects of age group [*F*_(1, 39)_ = 25.56, *p* < 0.001, η^2^_p_ = 0.40] and congruency [*F*_(1, 39)_ = 40.89, *p* < 0.001, η^2^_p_ = 0.51), and no significant interaction between age and congruency [*F*_(1, 39)_ = 1.36, *p* = 0.25]. For the comic completion task, ANOVAs for both accuracy rates and RTs also did not indicate any significant interaction between age and intention (Fs < 1). In addition, total AQ scores were similar for the older and young adults [*t*_(39)_ = 1.20, *p* = 0.24].

**Table 1:** Descriptive and comparative statistics for assessment data in older and young adults.

**Measures**	**Condition**	**Mean (SD)**	
		**Older**	**Young**
Years of education		13.25 (2.98)	13.31 (1.66)
SSS		5.45 (1.61)	6.48 (0.93)^#^
WM		8.00 (1.45)	10.76 (1.26)^***^
Inhibition acc.	Cong	100 (1)	100 (0)
	InCo	99 (2)	99 (2)
Inhibition RT	Cong	597 (114)	450 (43)
	InCo	678 (171)	506 (51)
ToM acc.	PhC	75 (21)	97 (5)
	Cint	75 (17)	94 (4)
ToM RT	PhC	2,629 (670)	1,835 (443)
	Cint	2,708 (676)	1,807 (423)
AQ		22.00 (6.27)	19.76 (5.64)

SSS, subjective socioeconomic status; WM, forward digit span task index; inhibition acc., accuracy rates (%) in the flanker task; inhibition RT, RTs (ms) in the flanker task; Cong, congruent condition; InCo, incongruent condition; ToM acc., accuracy rates (%) in the comic completion task; ToM RT, RTs (ms) in the comic completion task; PhC, physical causality condition; Cint, communicative intention condition; AQ, total scores of AQ questionnaire. Accuracy rates and RTs in both flanker task and comic completion task were analyzed with ANOVAs. AQ scores were compared with *T*-tests. And years of education, SSS, and WM span were compared with Wilcoxon tests for the data did not follow a normal distribution.

*** *p* < 0.001,

# p < 0.07.

### 2.2 Materials

In this study, we utilized a collection of dialogue scenarios to explore how individuals process various types of replies within a given context. Initially, we adapted 105 sets of written dialogue scenarios from Feng et al. (2017), and through a pretest (detailed in Supplementary materials), we carefully selected 90 sets that best aligned with our experimental objectives.

There were three scenarios in each set and each scenario corresponded to one particular experimental condition (see Table 2). Each dialogue scenario was designed to include a cover story and a round of question-and-reply dialogue. The cover story served as a brief introduction, setting the stage for the dialogue by providing necessary background information. For example, it might illustrate a scene at the company annual meeting where an employee walked down the stage and had a conversation with his colleagues. After the cover story, a yes/no question, which has only two possible answers (usually “yes” or “no”), followed naturally from the context that had been established. For instance, in the context of the annual meeting, the yes/no question could be “Is it easy to give a remarkable speech?”. The final part of each scenario was the critical reply, responding to the preceding question. In each set of scenarios, depending on the preceding question, a certain reply could serve as a direct reply (DR), a moderately indirect reply (MIR), and a highly indirect reply (HIR). The DR condition served as our baseline, where the reply directly addresses the question. In this condition, the speaker's intended meaning was the same as the literal meaning of the utterance. The MIR condition featured replies that were indirectly related to the question but still literally relevant, while the HIR condition featured replies that appeared irrelevant to the initial remarks but could be interpreted as deliberate indirect responses (Holtgraves, 1999). In these two conditions, the speaker's meaning was beyond the literal meaning of utterance, and the listener needed to infer the speaker's intention. For example, the utterance “Completing a great speech is very difficult” served as a direct reply to the question “Is it easy to give a remarkable speech?”, a moderately indirect reply to the question “Do you think my speech was good?”, and a highly indirect reply to the question “Were the audience members satisfied?”.

**Table 2:** An example of experimental conditions and a set of scenarios with translations. The vertical lines represent the segmentation of the replies.

**Condition**	**Cover story**	**Dialogue**
DR	公司年会上,一名 员工走下讲台,下 面是他和同事的对 话。	Q: 做一次好的演讲容易吗? Is it easy to give a remarkable speech? A: 完成|精彩的|演讲|非常|困难。
MIR	At the company annual meeting, an employee walked down the stage and had a conversation with his colleagues.	Q: 台下的听众感到满意吗 ? Do you think my speech was good? A: 完成|精彩的|演讲|非常|困难。
HIR		Q: 台下的听众感到满意吗? Were the audience members satisfied? A: 完成|精彩的|演讲|非常|困难。 Completing | (a) great | speech | (is) very | difficult.

These translations represent literal renderings of common Chinese expressions. The original Chinese expressions correspond to the following contextualized English sentences: “Isn't it hard to make a remarkable speech?”, “What do you think of my speech?', and “Was the audience satisfied with the speech?”.

It is important to note that in each set of materials, the three scenarios shared the same cover story and reply. All questions were designed to elicit answers that can be summarized with a “yes” or “no,” and each reply provided a clear answer to its preceding questions. In our given example, the reply “Completing a great speech is very difficult” essentially provided a “no” (No, the audience members were not satisfied) response to the question “Were the audience members satisfied?”. To ensure uniformity across all replies, we standardized the syntactic structure and syntactic relationships of all replies. Specifically, all replies were constructed in a subject-predicate format, where the subject consisted of three words or phrases, and the predicate was an adjective phrase with a modifier-head structure, including a degree adverb and an adjective. For example, the reply “完成 (Complete) 精彩的 (great) 演讲 (speech) 非常 (very) 困难 (difficult)” (Completing a great speech is very difficult) follows this structure, with “完成精彩的演讲” (Completing a great speech) as the subject and “非常困难” (very difficult) as the predicate. In our materials, the subject served as the topic of the reply, identifying what the sentence is about, while the predicate served as the comment, providing the new information or the main point of the sentence. Considering the structure of the reply in our experiment, we roughly viewed the first three phrases (the subject) as representing the early-to-mid stage of sentence unfolding. The last two phrases, which constituted the predicate of the sentence, were similarly considered to represent the late stage of sentence unfolding. A cover story included between 13 and 35 characters (mean length = 22.3, SD = 5.2), a yes-no question consisted of 5 to 16 Chinese characters (mean length = 10.0, SD = 2.0), and a reply, which is the critical utterance, contained 10 to 12 characters (mean length = 10.9, SD = 0.6).

To manipulate contextual relevance, latent semantic analysis (LSA; Deerwester et al., 1990) was employed. It was trained on an extensive Chinese dataset (the complete text of Chinese Wikipedia; https://dumps.wikimedia.org) to calculate the cosine similarity between the context, which consists of a cover story and a question, and the reply. The mean similarity results in the three conditions were analyzed by a repeated-measure ANOVA, which showed a significant main effect of condition, *F*_(2, 178)_ = 15.32, *p* < 0.001, η^2^_p_ = 0.15, and also a decreasing order of the similarity over DR (mean = 0.26, SD = 0.23), MIR (mean = 0.22, SD = 0.22) and HIR (mean = 0.17, SD = 0.17) conditions. The three conditions were significantly different to one another (ps ≤ 0.01, with a false discovery rate [FDR] adjustment to account for multiple comparisons). The findings, providing an objective measurement of the differences between the three conditions, in combination with abounding corpus data, proved that the experimental manipulation was valid.

### 2.3 Procedure

The reading comprehension task lasted approximately 30 min (see Supplementary materials for procedure diagram). All scenarios were divided into three lists following a Latin-square design. The scenarios in each list were pseudo-randomized such that no more than three consecutive scenarios belonged to the same condition, and no more than four consecutive scenarios required the same type of response. Each list divided further into two sessions to avoid participants' fatigue during the experiment. Before recording, all participants completed a practice phase following an instruction to familiarize themselves with the task.

Each trial started with a fixation (“+”), presenting at the center of the screen for a duration from 1 to 2 s. Then participants were presented with the cover story and question consecutively and were asked to read them at their own pace. When they had finished reading, participants pressed the spacebar on a keyboard to move to the next screen. The reply was presented segment by segment with each reply split into 5 segments, each containing a word or a phrase with 2 or 3 characters (see Table 2).^1^ Each segment was presented for 0.5 s with an interval of 0.5 s. The interval between the cover story introducing the background information and the screen presenting the question was 0.5 s, while that between the question and its reply was from 0.5 to 1 s. Participants were required to judge the speaker's intended meaning of the reply by making a binary choice of “yes” or “no” for the question after one to three trials. Specifically, they had to determine whether the speaker's meaning aligned with a “yes” or “no” response based on the provided contextual information. For example, in the dialogue “Were the audience members satisfied? Completing a great speech is very difficult,” the speaker's meaning of the reply is “No, the audience members were not satisfied,” which essentially provides a “no” response to the question. In the trials required for judgment, two words, “yes” and “no”, were presented on the left and right side of the screen respectively for 3 s after the presentation of the reply. Participants needed to respond as accurately and as quickly as possible by pressing the “F” or “J” key on a keyboard with the index finger of either their right or left hand. The selected answer on the screen turned blue after the response, and disappeared immediately or disappeared if any response was not recorded in 3 s.

After the EEG recording, a flanker task, a digital span task and a comic completion task were completed by each participant. Then a Chinese adaptation of the AQ questionnaire was filled out to assess individuals' social proficiency.

### 2.4 Data acquisition and analysis

We used Cognionics Quick-20 for EEG recordings in this experiment with 19 scalp electrodes, Fp1, Fp2, F7, F3, Fz, F4, F8, T7, C3, Cz, C4, T8, P7, P3, Pz, P4, P8, O1 and O_2_ based on a 10–20 system. Data obtained with Cognionics Quick-20 were recorded through an amplifier with a 0.01–100 Hz band-pass filter at a 500 Hz sample rate using the Cognionics Data Acquisition 2.0 software. The left earlobe (A1) worked as referential signals. The impedances were maintained at levels below 10 kΩ. Different events, including various experimental conditions and various components of scenarios, were marked in the EEG data using unique markers.

To analyze EEG data, a 0.5–40 Hz band-pass filter was used with the average of the whole brain as a reference. Independent component analysis (ICA) was measured to delete eye-movement artifacts for EEG data. We truncated the time period of 200 ms before the presentation onset of the first phrase of the reply to 5,000 ms after it for analysis. We used the 200 ms of EEG activity preceding first phrase onset as a baseline to measure the ERP data of each participant. To elaborate, each reply was divided into five phrases, with the first three phrases forming the subject (early-to-mid stage) and the last two phrases forming the predicate (late stage). Each phrase was displayed for 500 ms, followed by a 500 ms blank screen. This means that after one phrase appeared, the next phrase would be presented after a total of 1,000 ms (500 ms presentation + 500 ms blank). The complete presentation of the reply, which included all five phrases, thus took a total of 5,000 ms. This 5,000 ms period was the critical window for EEG recording, during which we captured the neural responses to the sequential presentation of the phrases. Nine electrodes (F3, Fz, F4, C3, Cz, C4, P3, Pz, P4) were selected for further analysis, as their coverage over frontal, central, and parietal regions is consistently implicated in generating N400/LPC effects during language comprehension (e.g., Kutas and Federmeier, 2011; Coulson and Lovett, 2010). Artifacts with the criteria of ± 100 μV were removed during data averaging. Thus, the number of trials in averaging was 26.1 under the DR condition, 25.9 under the MIR condition and 25.7 under the HIR condition for the older group, while that was 27.1 under the DR condition, 27.1 under the MIR condition, and 27.0 under the HIR condition for the young group. At least 18 trials were averaged under each condition among each participant. Analyses were conducted on the mean amplitude values in the following two time-windows: 300–450 ms, and 700–1000 ms after every phrase in the reply sentence, based on the observation of waveforms and previous studies.

The behavioral data and the ERP data were acquired and conducted by repeated-measures ANOVAs. Statistical significance was indicated when α < 0.05, with *p*-values adjusted by the Greenhouse-Geisser correction for violation of sphericity assumption when necessary and by FDR for multiple comparisons (Benjamini and Hochberg, 1995).

## 3 Results

### 3.1 Behavioral results

**Figure 1:**
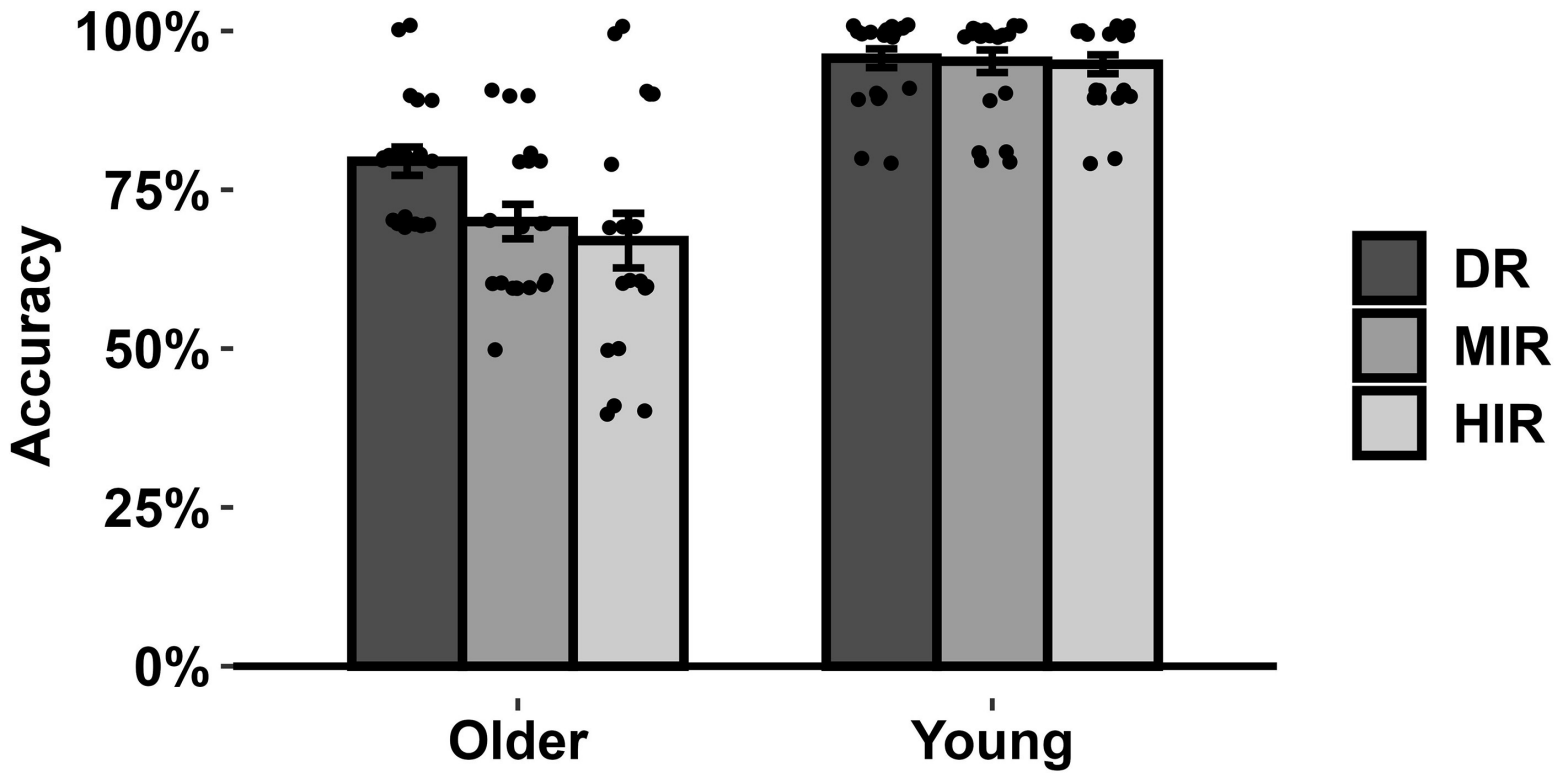
The mean accuracy rates from reading comprehension task in older and young adults. Error bars denote between-subject standard errors.

During EEG recording, task accuracy was also acquired and data analysis of task accuracy (see Figure 1) was conducted by repeated-measures ANOVA with age group as a between-subject factor and contextual relevance as a within-subject factor. Repeated-measures ANOVA revealed a significant main effect of group, *F*_(1, 39)_ = 80.52, *p* < 0.001, η^2^_p_ = 0.67, within older adults (mean ± SD = 67.0 ± 19.2%), compared to young adults (94.8 ± 6.8%), a significant main effect of contextual relevance, *F*_(2, 78)_ = 5.60, *p* = 0.008, η^2^_p_ = 0.13 and a significant interaction effect of group and contextual relevance, *F*_(2, 78)_ = 4.24, *p* = 0.023, η^2^_p_ = 0.10. Further analysis on simple-effect tests showed that effect of contextual relevance was significant in older adults, *F*_(2, 39)_ = 11.964, *p* < 0.001, η^2^_p_ = 0.38, but not in young adults [*F*_(2, 39)_ = 0.05, *p* = 0.95]. For older adults, the response accuracy in DR condition (79.5 ± 8.7%) was significantly higher than MIR (70.0 ± 10.5%) and HIR (67.0 ± 14.7%) conditions (ps < 0.001, Cohen's ds > 0.7), and there were no significant differences between MIR and HIR conditions (*p* = 0.37). These results suggested that the older adults had difficulties in interpreting indirect replies.

### 3.2 ERP results

**Figure 2:**
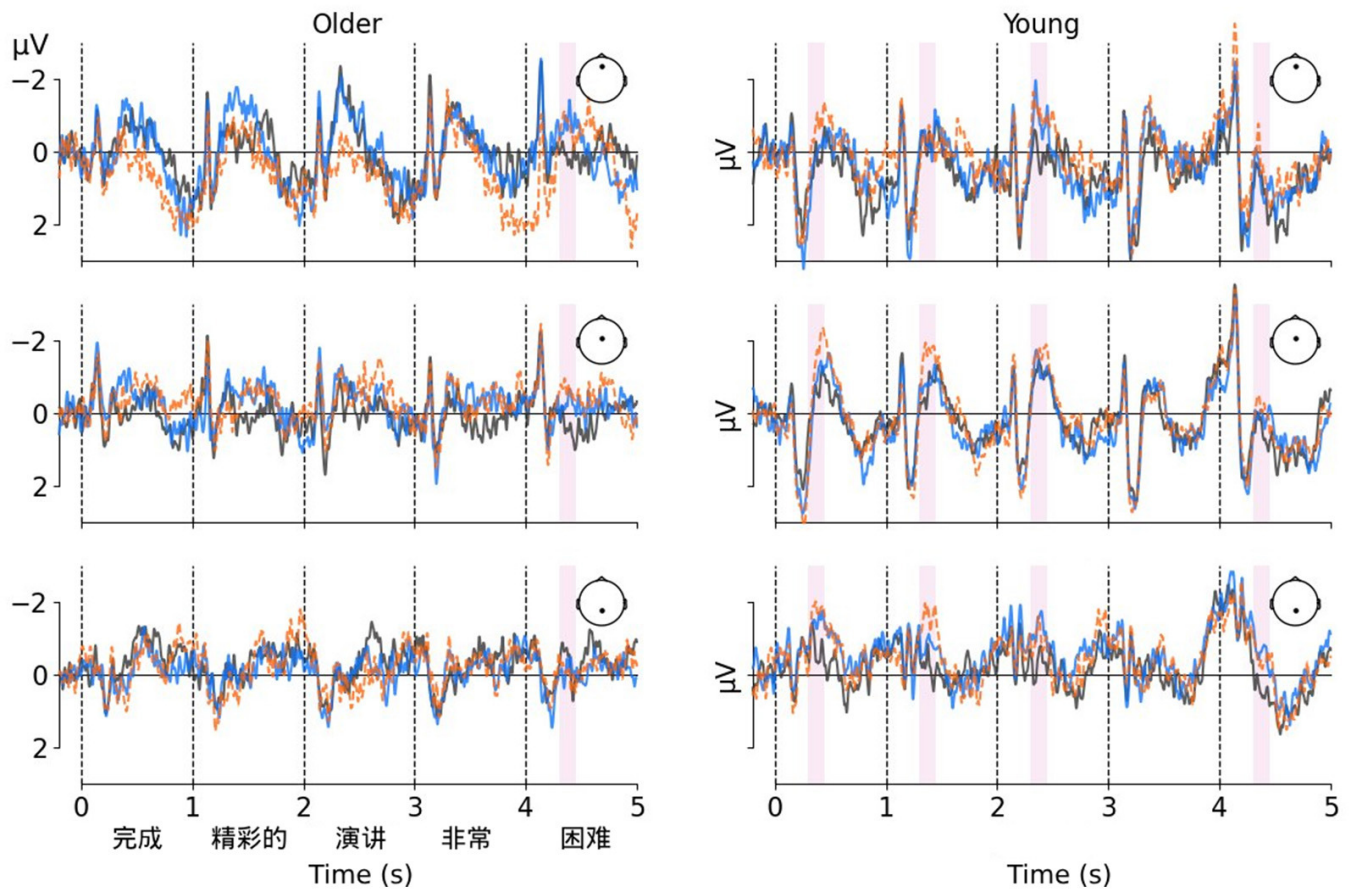
The correlation between working memory span and the N400 effects elicited by indirect reply for the second phrase.

The N400 amplitude was measured as the mean voltage in the 300–450 ms time window following the presentence of each phrase (see Figure 2). Repeated-measure ANOVAs were conducted respectively on the grand average ERP data during each time-window with contextual relevance (DR, MIR, and HIR conditions), hemisphere (left, center, and right), and region (front, center, and rear) as within-subject factors, and age group (the older and young adults) as a between-subject factor.

For the first phrase, an ANOVA for N400 revealed a significant main effect of hemisphere [*F*_(2, 78)_ = 6.09, *p* = 0.005, η^2^_p_ = 0.14], a significant two-way interaction between age group and region [*F*_(2, 78)_ = 5.35, *p* = 0.014, η^2^_p_ = 0.12], and a significant two-way interaction between contextual relevance and age group [*F*_(2, 78)_ = 3.48, *p* = 0.039, η^2^_p_ = 0.08]. No other effects were detected (Fs < 2). Simple-effect analyses were carried out to dissect the interaction between age group and contextual relevance. The effect of contextual relevance was significant in the young group [*F*_(2, 39)_ = 5.45, *p* = 0.008, η^2^_p_ = 0.22], with the amplitude decreasing in the order of DR (mean ± SE = −0.06 ± 0.13 μV), MIR (−0.10 ± 0.16 μV), and HIR (−0.67 ± 0.14 μV) condition, while not in the older group [*F*_(2, 39)_ = 0.18, *p* = 0.84]. *Post-hoc* tests for the young group showed that the amplitudes in HIR condition elicited were larger than both those in DR (*t* = −2.61, *p* = 0.019, Cohen's *d* = −0.21) and MIR (*t* = −3.05, *p* = 0.012, Cohen's *d* = −0.20) condition, with FDR correction for multiple comparisons.

For the second phrase, an ANOVA for N400 revealed a similar pattern with the first phrase. A main effect of contextual relevance [*F*_(2, 78)_ = 3.98, *p* = 0.028, η^2^_p_ = 0.09] and a two-way interaction between contextual relevance and age group [*F*_(2, 78)_ = 5.74, *p* = 0.007, η^2^_p_ = 0.13] was significant. Further analyses showed that the effect of contextual relevance was pronounced in the young group [*F*_(2, 39)_ = 11.39, *p* < 0.001, η^2^_p_ = 0.37], with the N400 amplitudes decreasing in the sequence of DR (−0.12 ± 0.11 μV), MIR (−0.20 ± 0.13 μV), and HIR (−0.82 ± 0.12 μV) conditions. In contrast, this effect was not observed in the older group [*F*_(2, 39)_ = 2.00, *p* = 0.15]. FDR correct *post-hoc* tests for the young group demonstrated that the amplitudes elicited in the HIR condition were significantly larger than those in both the DR (*t* = −3.51, *p* = 0.002, Cohen's *d* = −0.29) and MIR (*t* = −4.09, *p* < 0.001, Cohen's *d* = −0.26) conditions.

For the third phrase, an ANOVA for N400 also revealed a significant two-way interaction between contextual relevance and age group [*F*_(2, 78)_ = 3.28, *p* = 0.044, η^2^_p_ = 0.08]. Simple-effect analyses revealed that contextual relevance marginally significantly influenced the younger group's N400 amplitudes [*F*_(2, 39)_ = 2.89, *p* = 0.067, η^2^_p_ = 0.13], with a descending order from DR (−0.24 ± 0.18 μV) to MIR (−0.49 ± 0.19 μV) to HIR (−0.67 ± 0.16 μV) conditions. This effect was absent in the older group. *Post-hoc* tests for the young group confirmed that amplitudes in HIR condition were marginally larger than those in DR condition (*t* = −2.41, *p* = 0.063, Cohen's *d* = −0.18).

For the fourth phrase, neither the main effect of contextual relevance nor any interactions involving contextual relevance reached a significant level (Fs < 1.5).

For the fifth phrase, an ANOVA for N400 revealed a significant main effect of contextual relevance [*F*_(2, 78)_ = 9.10, *p* < 0.001, η^2^_p_ = 0.19], and no significant interaction between contextual relevance and other factors (Fs ≤ 2). *Post-hoc* tests further showed that amplitudes in both MIR (0.02 ± 0.10 μV; *t* = −3.05, *p* = 0.006, Cohen's *d* = −0.16) and HIR (−0.05 ± 0.09 μV; *t* = −3.67, *p* = 0.002, Cohen's *d* = −0.19) condition were significantly larger than those in DR (0.38 ± 0.09 μV) condition.

Thus, indirectness seemed to ramp up the N400 effect for the first three phrases in young adults, but this trend does not hold for older adults. For the final phrase, however, we see a strong N400 response in both age groups. In addition, considering the previous studies, we also tested late ERP components in the 700–1,000 ms time window for each phrase, but no statistically significant differences induced by contextual relevance were observed in any analysis in that time window.

### 3.3 The correlation between Individual differences and N400 effect

**Figure 3:**
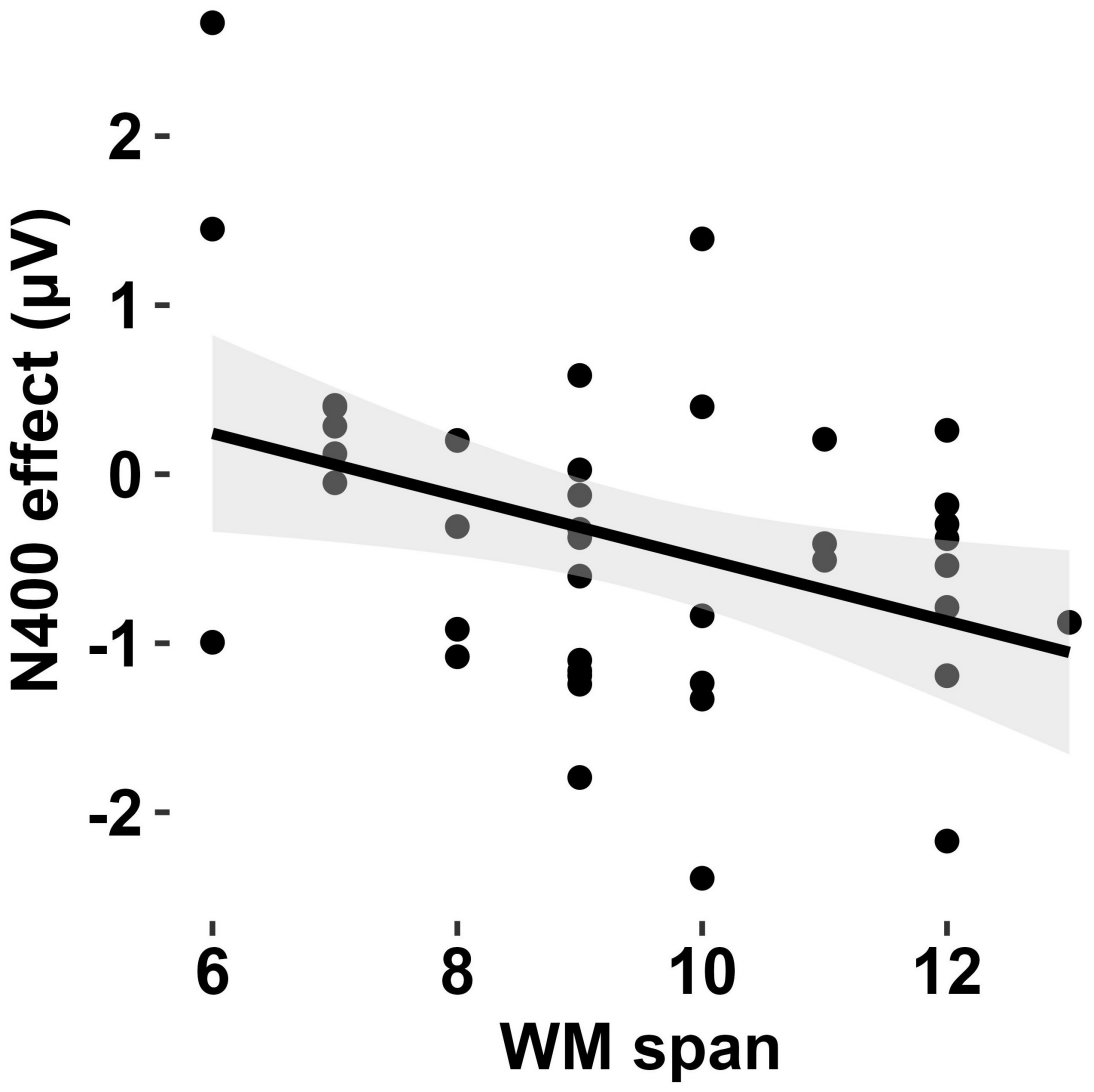
Grand-averaged ERP waveforms at the reply under the DR (in gray), MIR (in blue), HIR (in orange) conditions at midline electrodes (Fz, Cz and Pz). Data are shown for the older (left panels) and young (right panels) adults. The shadows represent the time window of the N400 effects elicited by indirect reply in the current study.

To further investigate the sources of variability that could clarify age-group differences in N400 effects of contextual relevance during comprehending indirect replies, we conducted Pearson correlation analyses to explore individual differences in the size of N400 effect, which was calculated as the difference in mean amplitude between HIR and DR condition in the first three phrases. Results showed that only the WM span of individuals was significantly correlated with N400 effect for the second phrases (*r* = −0.37, *p* = 0.02; see Figure 3). To account for potential confounding effects of educational attainment (years) and subjective socioeconomic status, a partial correlation analysis was performed. After controlling for these covariates, a statistically significant inverse relationship persisted between WM span and N400 effect magnitude (*pr* = −0.39, *p* = 0.01). In addition, such correlation also held true in the older group for the second phrases (*r* = −0.46, *p* = 0.04). That is, adults with long working memory span were more likely to have shown larger N400 effects during indirect reply comprehension.

## 4 Discussion

This research aimed to explore whether there are any differences, and if so, what kind of differences exist, between the elderly and the young in terms of indirect reply comprehension. Behavioral results revealed that unlike young adults, the elderly performed worse under indirect reply conditions than under direct reply conditions. This finding confirms the existence of age-related declines in comprehending indirect speech, aligning with previous research on the aging of communicative-pragmatic abilities (Bambini et al., 2021; Baraldi and Domaneschi, 2024; Rothermich et al., 2021). More importantly, EEG data indicated that there were also distinctions in the time course of indirect reply comprehension between the older and young adults. For both older and young adults, indirect replies elicited a larger N400 effect in the later stages compared to direct replies. However, among young adults, indirect replies triggered increased N400 effects than direct replies during the early and middle stages of reply presentation, a distinction which was not significant in the older adults.

First, we found that indirect replies elicited enhanced N400 effects for the first three phrases and the fifth phrase in young adults. This supports the idea that pragmatic factors have an early and sustained influence on utterance understanding. N400 is generally considered as a typical ERP component, reflecting the processing of semantic retrieval and integration in language comprehension (Lau et al., 2008; Kutas and Federmeier, 2011). An N400 effect would be elicited and observed when individuals encounter words that are difficult to integrate into the context of the sentence, such us verb-noun mismatch (Hahne and Friederici, 2002; Zhang et al., 2010), adjective-noun mismatch (Hagoort, 2003; Prior and Bentin, 2006), classifier-noun mismatch (Zhou et al., 2010), semantic plausibility combined with unexpectedness (Camblin et al., 2007; Federmeier and Kutas, 1999; Wlotko and Federmeier, 2007), or contradiction with world knowledge (Hagoort et al., 2004; Hald et al., 2007). Moreover, the N400 effect has been shown to be sensitive not just to sentence-level semantics but also to discourse-level processing (Nieuwland and Van Berkum, 2006; van Berkum et al., 1999). When a sentence contains pragmatic anomalies that violate contextual expectations, the N400 effect becomes more pronounced. This amplified response reflects the additional cognitive processing required to resolve these interpretative anomalies (Jiang et al., 2013; Nieuwland et al., 2010). Jiang et al. (2013) focused on the Chinese “lian…dou…” construction, akin to the English word “even”, and investigated how pragmatic constraints based on constructions influence sentence processing. Both the pragmatically incongruent (e.g., “Zhanghong can hear even such loud sounds clearly…”) and underspecified (“Zhanghong can hear even such a kind of sound clearly…”) conditions elicited larger N400 effects than the congruent condition (“Zhanghong can hear even such tiny sounds clearly…”), which reflecting the increased difficulty in immediately integrating the current event into the conventional meaning representation generated by “lian…dou…” construction. In addition, studies on scalar implicature revealed that pragmatically underinformative statements (e.g., “Some people have lungs...”), compared to pragmatically appropriate statements (“Some people have pets…”), elicited a greater N400 effect at the critical word (pets/lungs), whether it is when the quantity expression of underinformative statements is pragmatically incongruent with world knowledge (Nieuwland et al., 2010) or immediate pictorial contexts (Spychalska et al., 2016; Zhao et al., 2021). More relevantly, indirect replies and requests have been found to elicit larger N400 effects (Coulson and Lovett, 2010; Guo et al., 2023; Zhang et al., 2023), reflecting more difficulty in semantic processing. Therefore, the N400 enhancement throughout the entire utterance in the present study would suggest that young adults exert greater effort on semantic processing from the very beginning stage of indirect reply comprehension, which is crucial to bridging the semantic gap between the current content and its context, and for establishing efficient semantic integration.

Nevertheless, such N400 effects were absent for the first three phrases in the elderly, which might be a major factor contributing to their poorer behavioral performance in understanding conversational implicatures requiring pragmatic inference. Previous studies have shown that the N400 effect associated with semantic processing was modified by healthy aging (Joyal et al., 2020; Wlotko et al., 2010). Compared to young adults, older adults showed significantly smaller N400 response to semantically incongruent or unexpected target words than congruent or expected ones in sentence processing, which may suggest that elderly individuals have a diminished ability to rapidly use predictive information from contexts (e.g., Federmeier and Kutas, 2005; Payne and Federmeier, 2018; Wlotko et al., 2012). In other words, older individuals are less likely to employ a pre-activation comprehension strategy in a similar manner as younger individuals (Broderick et al., 2021). Therefore, as observed in the present study, the absent or reduced N400 effect at the early and middle stage of indirect reply comprehension in older adults may reflect the untimeliness in semantic enrichment and integration brought about by aging during pragmatic processing. Meanwhile, like the young group, a larger N400 response was observed for the final phrase in the older group. This finding presumably demonstrates that older adults tend to perform the overall integration of utterance content in sentence-final position (van Berkum et al., 2003), to generate implicate meanings. It should be noted that the N400 effect triggered by indirect replies was not observed in the fourth phrase, which may be due to the sentence structure of the reply utterances. In all experimental materials, the fourth phrase of the reply was always a degree adverb from a relatively small set, which made this word very predictable and easy to integrate into the existing discourse representation across all experimental conditions.

Furthermore, our study showed that older participants scored significantly lower in WM span than younger ones, and individual differences in WM capacity could modulate the N400 indirectness effect at the early stage of reply comprehension when controlling statistically for the educational attainment and subjective socioeconomic status. More specifically, older adults with high WM capacity performed neural processing patterns more like the young. Previous studies with young populations found that individuals' WM capacity is related to the size of the enhanced N400 effect elicited by semantic anomalies (Van Petten et al., 1997; Yang et al., 2020) and semantic ambiguity (Gunter et al., 2003). When it comes to heath aging, older adults exhibit a reduced and delayed N400 effect in semantic processing, coinciding with their diminished WM capacity. Federmeier and Kutas (2005) examined how older and young adults used contextual information during the process of language comprehension. The results showed that both older and younger groups showed reduced N400 effect for strongly constraining contexts compared to weak ones, whereas, for older adults, this effect was significantly smaller and later. At the same time, verbal WM span has been identified as a robust predictor of age-related changes in the N400 constraint effect. Hence, the decline in WM may leave older adults with insufficient ability to effectively and rapidly utilize linguistic contextual information in language comprehension (Burke and Shafto, 2008). The findings of Zhang et al. (2021) further support the impact of WM capacity on the comprehension of indirect replies. Although their study did not uncover an N400 indirectness effect as seen in our study and previous studies on indirect reply comprehension (Guo et al., 2023; Zhang et al., 2023), it indeed showed that individuals with low WM span did not exhibit earlier electrophysiological component differences (P200 and P300 time window) elicited by indirect replies at the final phrase, compared to those with high WM span. Behaviorally, WM was also found to reliably predict the performance across multiple pragmatic tasks with healthy aging (Bambini et al., 2021). Therefore, one possible explanation of our finding is that the aging of pragmatic inference may stem from the decline in WM capacity, facilitated by the constraints that WM capacity imposes on the immediate semantic enrichment and integration during interpreting a given speaker's meaning. That is, older adults performed the reading comprehension task in the indirect reply condition with relatively low accuracy because their limited WM span is less likely to support a comprehension strategy similar to that of the young, where upcoming words are quickly processed and integrated into the discourse representation.

It is noteworthy that the result pattern of the current study is different from previous ERP studies on indirect reply comprehension. Actually, as mentioned above, the findings of prior studies have also been inconsistent (Guo et al., 2023; Zhang et al., 2021, 2023). In prior studies, the identical critical utterance (e.g., I | really | have too many) could be interpreted as either an indirect reply or a direct reply according to its context. The results demonstrated indirect replies elicited an enhanced sustained positivity (P200, P300, and LPC) at the final phrase, as observed in Zhang et al. (2021), or a larger N400 or LPC at the final phrase, as reported in Guo et al. (2023), or a larger negativity at the second phrase and a greater N400 at the final phrase, as reported in Zhang et al. (2023). Differently from the present study, previous studies have revealed ERP indirectness effects that are primarily manifested at the end of utterances, and most have identified late positive or negative components. These discrepancies may be due to the structure of the critical utterances in our stimuli materials. For each scenario, the critical utterance adheres to a subject-predicate structure, with the subject comprising three phrases. The subject captures the topic of the utterance, from which it can be noticed whether the reply is directly related to the preceding question. Such material design prevents the process of indirect reply comprehension from being compressed into a single phrase; instead, it is incremental as the entire sentence unfolds. Thus, the processing of pragmatic inference, especially the processing of semantic activation and integration necessary for constructing discourse representation, could occur at the first three phrases of the utterance. Also, the N400 indirectness effect observed for the final phrase could be caused by sentence-final “wrap-up” processes (Hagoort, 2003).

## 5 Conclusion

Our study explored the behavioral and neural underpinnings of comprehending indirect replies in both older and young adults. Behaviorally, our findings showed that older adults had difficulty with indirect reply comprehension. ERP data further indicated that indirect replies elicited an increased N400 response in both age groups during the final phases of the reply compared to direct replies. However, young adults showed an enhanced N400 effect for indirect replies compared to direct ones in the early and middle stages of reply representation, while such N400 indirectness effects failed to be observed among older adults. This suggests that the pragmatic inference capabilities of older individuals may be diminished relative to younger ones, potentially due to challenges in immediacy and dynamically enrichment of the semantic content of ongoing speech. Our findings underscore the impact of aging on the time course of pragmatic processing during language comprehension, offering insights into cognitive decline associated with aging.

## Data Availability

The raw data supporting the conclusions of this article will be made available by the authors, without undue reservation.

## Footnotes

^1^In this study, each reply sentence was divided into five segments, with each segment containing a Chinese word or phrase. For example, the sentence “完成精彩的演讲非常困难 (Completing a great speech is very difficult)” was broken down into five segments: “完成” (complete), “精彩的” (great), “演讲” (speech), “非常” (very), and “困难” (difficult). Here, “完成,” “演讲,” “非常,” and “困难” are typical Chinese words, while “精彩 的 ” is a phrase in Chinese, which functions grammatically as an attributive, composed of the adjective “精 彩” (great) and the structural particle “的”. The presence of “的” made the sentence smoother and more in line with the linguistic intuition of native Chinese speakers. Given that it was common in the experimental materials for a segment to be either a word or a phrase (like “精彩的”), to maintain rigorous expression, we would uniformly refer to these segments as “phrases” in the subsequent text.
