# Supplementary material for: Time course of indirect reply comprehension in the young and older adults: an event-related potential study
Source: Front Psychol. 2025 Jul 10;16:1578192. doi: 10.3389/fpsyg.2025.1578192 (PMC12287003; doi:10.3389/fpsyg.2025.1578192)
Supplement: Supplementary file 1 [file Supplementary_file_1.docx]

*Supplementary Materials* for

Time course of indirect reply comprehension in the young and older adults: An event-related potential study

Wangshu Feng^a^, Xiaokun Zhang^b^, Weijuan Wang^c^ and Lin Fan^b^

*^a^ Artificial Intelligence and Human Languages Lab, Beijing Foreign Studies University, Beijing 100089, China;*

*^b^ National Research Centre for Foreign Language Education, Beijing Foreign Studies University, Beijing 100089, China;*

*^c^ School of Foreign Languages, Qingdao University of Science and Technology, Qingdao, China*

## *Norming Study of the Experimental Materials*

To ensure the quality of the experimental materials and the success of the experimental manipulation, we conducted a pretest before the EEG experiment. Thirty college students (15 females, mean age 22.4 ± 4.0 years) who had not participated in the EEG recording were recruited from Beijing Foreign Studies University. Using a Latin-square procedure, 105 scenarios were divided into three experimental lists, with each list being scored by 10 participants.

In this pretest, participants were presented with one list of scenarios. For each trial, they read the scenario carefully and make a binary judgment about the intended meaning of the speakers’ replies. Specifically, they had to determine whether the speaker’s meaning aligned with a “yes” or “no” response based on the provided contextual information. The term “speaker’s meaning” refers to the true intention or message that the speaker aims to convey through their utterance. In the context of our study, it is the underlying intent that participants need to infer from the dialogue, which may not always be explicitly stated. For example, in the dialogue “Were the audience members satisfied? Completing a great speech is very difficult,” the speaker’s meaning of the reply is “No, the audience members were not satisfied,” which essentially provides a “no” response to the question. This step was largely consistent with the requirements of the reading comprehension task in the EEG recording. It was crucial for assessing the clarity of the dialogues in each condition, as it enabled us to determine the comprehension of each experimental material by the individuals. Following this, participants assessed the indirectness of the replies using a 7-point visual analog scale, where “1” indicated the most direct reply with the lowest level of indirectness, and “7” represented the most indirect reply with the highest level of indirectness. This method allowed us to evaluate how participants perceived the degree of indirectness in the speakers’ replies across various conditions.

We used the participants’ binary judgments as the basis for our assessment to ensure that the dialogues in various conditions were clear and unambiguous. If participants consistently made accurate binary judgments, it indicated that the replies were clear and effectively conveyed the intended meaning regarding their preceding questions. Conversely, if there was significant variability in participants’ responses, it suggested that the dialogue might be ambiguous or unclear, prompting us to exclude those scenarios from the final experimental materials. To reduce the impact of ambiguity in interpreting dialogues, we extracted 90 sets of scenarios after deleting of 15 sets where speakers’ meanings in one or more scenarios were differently perceived by two or more participants.

For the analysis of reaction times and rating scores on correct responses, repeated measured ANOVAs were conducted separately with contextual relevance as within-subject factor. There was a significant main effect of contextual relevance, *F* (2, 58) = 30.56, *p* < 0.001, *η²_p_* = 0.51, with an increased time duration in the DR (2016 ± 347 ms), MIR (2113 ± 357 ms) and HIR (2257 ± 370 ms) conditions. Pairwise comparison revealed significant differences between the three conditions, i.e., DR, MIR, and HIR replies (*p*s ≤ 0.005, with FDR correction). Consistent with the above-mentioned LSA, there was a main effect of contextual relevance on indirectness rating scores, *F* (2, 58) = 127.4, *p* < 0.001, with an increase in DR (1.95 ± 0.50), MIR (4.14 ± 0.71), and HIR (4.58 ± 0.79) conditions. Pairwise comparison showed significant differences between the DR, MIR, and HIR replies (*p*s < 0.02, with FDR correction). These results indicate that the manipulation of contextual relevance was successful.

## *The experimental* *procedure diagram for the reading comprehension task*


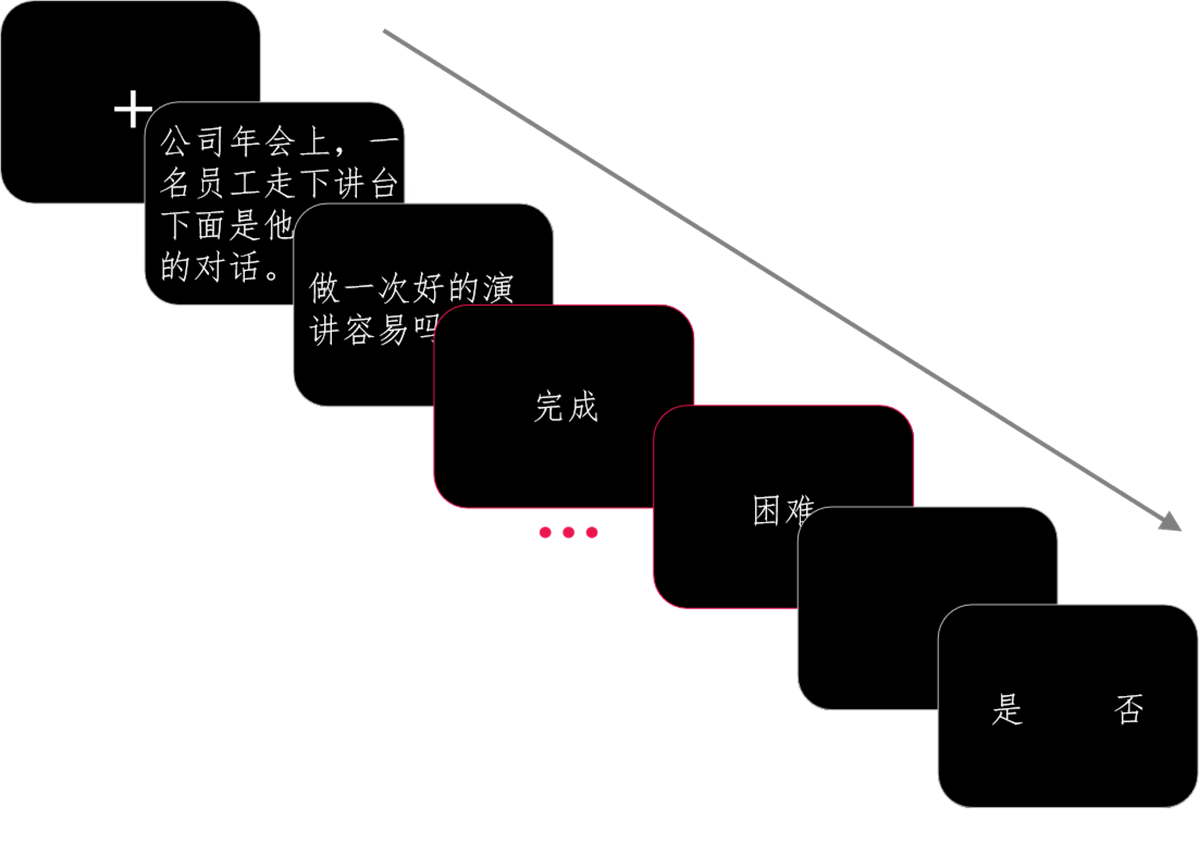


Supplementary Figure 1. In each trial of the reading comprehension task, the fixation point, cover story, question, and the five phrases of the reply were presented sequentially in the center of the screen. In 1/3 trials, the screen displayed options for “yes” and “no,” requiring participants to judge the speaker’s meaning of the reply. The red boxes mark the critical screens for EEG recording.
